# Social conditions of becoming homelessness: qualitative analysis of life stories of homeless peoples

**DOI:** 10.1186/s12939-017-0646-3

**Published:** 2017-08-22

**Authors:** Mzwandile A. Mabhala, Asmait Yohannes, Mariska Griffith

**Affiliations:** 1 Faculty of Health and Social Care, Department of Public Health and Wellbeing, University of Chester, Riverside Campus, Chester, CH1 1SL UK; 2 Mount Sinai, Department of Surgery, Ambulatory Surgery Centre, 5 East 98th Street, 14th Floor, Box 1259, New York, NY 10029-6574 USA; 3 Department of Public health and Wellbeing, University of Chester, Riverside Campus, Chester, CH1 1SF UK

## Abstract

**Background:**

It is increasingly acknowledged that homelessness is a more complex social and public health phenomenon than the absence of a place to live. This view signifies a paradigm shift, from the definition of homelessness in terms of the absence of permanent accommodation, with its focus on pathways out of homelessness through the acquisition and maintenance of permanent housing, to understanding the social context of homelessness and social interventions to prevent it.

However, despite evidence of the association between homelessness and social factors, there is very little research that examines the wider social context within which homelessness occurs from the perspective of homeless people themselves. This study aims to examine the stories of homeless people to gain understanding of the social conditions under which homelessness occurs, in order to propose a theoretical explanation for it.

**Method:**

Twenty-six semi-structured interviews were conducted with homeless people in three centres for homeless people in Cheshire North West of England.

**Results:**

The analysis revealed that becoming homeless is a process characterised by a progressive waning of resilience capacity to cope with life challenges created by series of adverse incidents in one’s life. The data show that final stage in the process of becoming homeless is complete collapse of relationships with those close to them. Most prominent pattern of behaviours participants often describe as main causes of breakdown of their relationships are:

1. engaging in maladaptive behavioural lifestyle including taking drugs and/or excessive alcohol drinking
2. Being in trouble with people in authorities.

**Conclusion:**

Homeless people describe the immediate behavioural causes of homelessness, however, the analysis revealed the social and economic conditions within which homelessness occurred. The participants’ descriptions of the social conditions in which were raised and their references to maladaptive behaviours which led to them becoming homeless, led us to conclude that they believe that their social condition affected their life chances: that these conditions were responsible for their low quality of social connections, poor educational attainment, insecure employment and other reduced life opportunities available to them.

## Background

It is increasingly acknowledged that homelessness is a more complex social and public health phenomenon than the absence of a place to live. This view signifies a paradigm shift, from the definition of homelessness in terms of the absence of permanent accommodation [1–5], with its focus on pathways out of homelessness through the acquisition and maintenance of permanent housing [6], to understanding the social context of homelessness and social interventions to prevent it [6].

Several studies explain the link between social factors and homelessness [6–10]. The most common social explanations centre on seven distinct domains of deprivation: income; employment; health and disability; education, skills and training; crime; barriers to housing and social support services; and living environment [11]. Of all forms, income deprivation has been reported as having the highest risk factors associated with homelessness [7, 12–14]: studies indicate that people from the most deprived backgrounds are disproportionately represented amongst the homeless [7, 13]. This population group experiences clusters of multiple adverse health, economic and social conditions such as alcohol and drug misuse, lack of affordable housing and crime [10, 12, 15]. Studies consistently show an association between risk of homelessness and clusters of poverty, low levels of education, unemployment or poor employment, and lack of social and community support [7, 10, 13, 16].

Studies in different countries throughout the world have found that while the visible form of homelessness becomes evident when people reach adulthood, a large proportion of homeless people have had extreme social disadvantage and traumatic experiences in childhood including poverty, shortage of social housing stocks, disrupted schooling, lack of social and psychological support, physical, sexual, and emotional abuse, neglect, dysfunctional family environments, and unstable family structures, all of which increase the likelihood of homelessness [10, 13, 14].

Furthermore, a large body of evidence suggests that people exposed to diverse social disadvantages at an early age are less likely to adapt successfully compared to people without such exposure [9, 10, 13, 17], being more susceptible to adopting maladaptive coping behaviours such as theft, trading sex for money, and selling or using drugs and alcohol [7, 9, 18, 19]. Studies show that these adverse childhood experiences tend to cluster together, and that the number of adverse experiences may be more predictive of negative adult outcomes than particular categories of events [17, 20]. The evidence suggests that some clusters are more predictive of homelessness than others [7, 12]: a cluster of childhood problems including mental health and behavioural disorders, poor school performance, a history of foster care, and disrupted family structure was most associated with adult criminal activities, adult substance use, unemployment and subsequent homelessness [12, 17, 21]. However, despite evidence of the association between homelessness and social factors, there is very little research that examines the wider social context within which homelessness occurs from the perspective of homeless people themselves.

This paper adopted Anderson and Christian’s [18] definition, which sees homelessness as a ‘function of gaining access to adequate, affordable housing, and any necessary social support needed to ensure the success of the tenancy’. Based on our synthesis of the evidence, this paper proposes that homelessness is a progressive process that begins at childhood and manifests itself at adulthood, one characterised by loss of the personal resources essential for successful adaptation. We adopted the definition of personal resources used by DeForge et al. ([7], p. 223), which is ‘those entities that either are centrally valued in their own right (e.g. self-esteem, close attachment, health and inner peace) or act as a means to obtain centrally valued ends (e.g. money, social support and credit)’. We propose that the new paradigm focusing on social explanations of homelessness has the potential to inform social interventions to reduce it.

In this study, we examine the stories of homeless people to gain understanding of the conditions under which homelessness occurs, in order to propose a theoretical explanation for it.

## Method

The design of this study was philosophically influenced by constructivist grounded theory (CGT). The aspect of CGT that made it appropriate for this study is its fundamental ontological belief in multiple realities constructed through the experience and understanding of different participants’ perspectives, and generated from their different demographic, social, cultural and political backgrounds [22]. The researchers’ resulting theoretical explanation constitutes their interpretation of the meanings that participants ascribe to their own situations and actions in their contexts [22].

The stages of data collection and analysis drew heavily on other variants of grounded theory, including those of Glaser [23] and Corbin and Strauss [24].

### Setting and sampling strategy

The settings for this study were three centres for homeless people in two cities (Chester and Crewe) in Cheshire, UK. Two sampling strategies were used in this study: purposive and theoretical. The study started with purposive sampling and in-depth one-to-one semi-structured interviews with eight homeless people to generate themes for further exploration.

One of the main considerations for the recruitment strategy was to ensure that the process complies with the ethical principles of voluntary participation and equal opportunity to participate. To achieve this, an email was sent to all the known homeless centres in the Cheshire and Merseyside region, inviting them to participate. Three centres agreed to participate, all of them in Cheshire – two in Chester and one in Crewe.

Chester is the most affluent city in Cheshire and Merseyside, and therefore might not be expected to be considered for a homelessness project. The reasons for including it were: first, it was a natural choice, since the organisations that funded the project and the one that led the research project were based in Chester; second, despite its affluence, there is visible evidence of homelessness in the streets of Chester; and third, it has several local authority and charity-funded facilities for homeless people.

The principal investigator spent 1 day a week for 2 months in three participating centres, during that time oral presentation of study was given to all users of the centre and invited all the participants to participate and written participants information sheet was provided to those who wished to participate. During that time the principal investigator learned that the majority of homeless people that we were working with in Chester were not local. They told us that they came to Chester because there was no provision for homeless people in their former towns.

To help potential participants make a self-assessment of their suitability to participate without unfairly depriving others of the opportunity, participants information sheet outline criteria that potential participants had to meet: consistent with Economic and Social Research Council’s Research Ethics Guidebook [25], at the time of consenting to and commencing the interview, the participant must appear to be under no influence of alcohol or drugs, have a capacity to consent as stipulated in England and Wales Mental Capacity Act 2005 [26], be able to speak English, and be free from physical pain or discomfort.

As categories emerged from the data analysis, theoretical sampling was used to refine undeveloped categories in accordance with Strauss and Corbin’s [27] recommendations. In total 26 semi-structured interviews were carried out. Theoretical sampling involved review of memos or raw data, looking for data that might have been overlooked [27, 28], and returning to key participants asking them to give more information on categories that seemed central to the emerging theory [27, 28].

The sample comprised of 22 male and 4 female, the youndgest participant was 18 the eldest was 74 years, the mean age was 38.6 years. Table 1 illustrates participant’s education history, childhood living arrangements, brief participants family and social history, emotional and physical health, the onset of and trigger for homelessness.

**Table 1:** Participants’ demographic information

Pseudonym	Biographical data	Education history	Childhood living arrangements	Summary of participant’s family and social history	Emotional and physical health	Onset of and trigger for homelessness
Ruddle	Age: 60 Gender: M Address: Flat for homeless people	Age of leaving school: 16 Type of school: local school Performance: very poor Behaviour: shared accommodation	Grew up with both parents	Dad died and house sold when participant was 15 Disruptive family life	‘If I need see a doctor I just walk into A&E’	Both parents had died by the time he was 15
Ian	Age: 74 Gender: M Address: Street	Age of leaving school: 16 Type of school: local school Performance: very poor Behaviour: in trouble with school authorities	Grew up with both parents	Normal working class family	No health problems	Breakdown of relationship
Patrick	Age: 64 Gender: M Address: Flat	Age of leaving school: 16 Type of school: local school Performance: very poor Behaviour: disruptive behaviour	Raised by both parents	Left home at age 15 In prison twice for theft Both parents never worked, both parents alcoholics	Bad heart, kidney and liver	Became homeless after release from prison
Lee	Age: 35 Gender: M Address: Street	Age of leaving school: 15 Type of school: mainstream school, ‘a rough school’ Performance: ‘I was in lowest set’ Behaviour: truancy and challenging authorities	Raised in children’s care	Parents split up when he was 13 years old Chaotic family life ‘I was 15 when I left school’ Arrested at 18, followed by several episodes of time in prison	‘[I’ve been to] A&E that many times because of accident[s]’	‘You are out, she couldn’t cope but I was only 17’ ‘She kicked me out again when I was 18’
David	Age: 31 Gender: M Address: Street	Age of leaving school: 16 Type of school: kicked out of high school, went to special school Performance: very poor Behaviour: disruptive behaviour	Foster care	Neglected by his mum	‘Hepatitis C in my liver’ Depression	Became homeless soon after leaving foster care at 8. Maladaptive behaviour, excessive alcohol use
Marco	Age: 46 Gender: M Address: Street	Age of leaving school: 16 Type of school: ‘moved several schools’, ‘finished in school for the naughty boys’ Performance: very unpleasant school experience Behaviour: engaged in several fights, bullied	Boarding school for boys with challenging behaviour	Very abusive family – physical and emotional Multiple failed relationships	Memory problem ‘I am on medication that doesn’t permit me to use any machinery’ Bad back, ruptured disc and nerve damage	Breakdown of relationship with partner
Alvin	Age: 39 Gender: M Address: Hostel for homeless people	Age of leaving school: 16 Type of school: local school Performance: very poor Behaviour: in trouble with school authorities		Dad died when he was 13 Trained as bricklayer, after recession worked in factory Started using drugs and alcohol	Depression Anxiety disorder	Marriage breakdown due to financial stress and alcohol addiction
Barry	Age: 43 Gender: Male Address: Hostel for homeless people	Age of leaving school: Law degree at University of Oxford Type of school: local school Performance: very poor Behaviour: in trouble with school authorities	Family home; middle class family	Joined the army at 18 Went to university after discharge from the army	No medical problems	Kicked out by parents when found out he was gay. Relationship breakdown Evicted while at the hospital
Clarke	Age: 18 Gender: M Address: Hostel for homeless people	Age of leaving school: 16 Type of school: local school Performance: very poor Behaviour: in trouble with school authorities	Family home, raised by mum and stepdad	Bullied at school Physically abused by stepdad Grand-dad, who he was very close to, died when was 16 Left home at 16. Unable to get council accommodation because he under 21. Lived in the street for two years	‘Feel low all the time’	Breakdown of child-parent relationship Physical abuse by stepdad Excessive alcohol use
Danny	Age: 44 Gender: M Address: Hostel for homeless people	Age of leaving school: 16 Type of school: local school Performance: very poor Behaviour: in trouble with school authorities	Raised by both parents	‘I left school at sixteen, straight away I got married and had children. I was married for seventeen years. I was a roofer for ten years. When marriage broke up I turned into alcoholic. I moved back with my parents. It got really out of control. I was getting in trouble with police’	Depression Anxiety disorder	Marriage breakdown Alcohol and drugs ‘I was getting in trouble with police’
Emily	Age: 23 Gender: F Address: Hostel for homeless people	Age of leaving school: 16 Type of school: local school Performance: very poor Behaviour: challenging behaviour	Single parent	Labelled as naughty child at school, ran away from home at 16. Lived with friends on night-by-night basis Had a job in Morrisons [supermarket] when 18, dismissed due to bad behaviour Kicked out of several hostels	ADHD	Breakdown of relation with mother Use of drugs and alcohol
Finn	Age: 24 Gender: M Address: Hostel for homeless people	Age of leaving school: 16 Type of school: ‘moved around many schools around the country’ Performance: very poor Behaviour: numerous fights at school	Single parent; transient	Single mum with several partners Moved in with brother at 16 Secured a job in convinience store	Depression	Kicked out by brother due to socially unacceptable lifestyle
Goeff	Age: 32 Gender: M Address: Hostel for homeless people	Age of leaving school: 16 Type of school: local school Performance: very poor Behaviour: in trouble with school authorities	Family home; raised by both parents	‘Parents died at young age. Lived with sister for a bit, fell out with her and ended up in the street. Been in the street for about 14 years’ ‘It started by going to jail at 16, I was in and out of jail for the past 16 years’		
Gary (John)	Age: 35 Gender: M Address: Hostel for homeless people	Age of leaving school: 16 Type of school: mainstream school Performance: very poor Behaviour: in trouble with school authorities	Raised by mum and stepdad until age 11	‘I worked as a joiner. I lost both my parents at a very young age. I lost my dad when I was four and lost my mum when I was eleven. Raised by my nan until the age of sixteen’ ‘I went to jail when I was 16 years in Liverpool, spent two years there’ ‘I have been assaulted that many times, that is where anxiety comes from’	‘I was depressed. I suffer from anxiety and depression’ Alcoholism Epilepsy	Alcoholism Relationship broke down Ended up on the street
Guy	Age: 26 Gender: M Address: Hostel for homeless people Northwich	Age of leaving school: 16 Type of school: mainstream school Performance: ‘I left with no GCSEs’ Behaviour: challenging behaviour	Dad was in the military	‘I used to smoke a lot of weed. I worked for Network Rail’ Jailed for drug possession. Since leaving school he has completed six jail sentences for motorbike theft	No health issues	‘I used to smoke a lot of weed and spice’ ‘My parent kicked me out of the house while I was jail’
Henry	Age: 29 Gender: M Address: Street Northwich	Age of leaving school: 15 Type of school: boarding school for pupils with emotional and behavioural difficulties Performance: very poor Behaviour: aggressive and disrruptive	Raised by stepdad and mum	‘I’ve NVQ 2 in electric, NVQ 2 in cooking, NVQ 1 plumbing’ ‘My mum was phyically abused by my stepdad, he used to beat her up. When they split up, my mum was taken to the care home where they used to look after her’ ‘I was in jail when I was sixteen’	Asthma Anxiety Digestive disorder Suicidal Paranoia through smoking cannabis	Physically abused by mum and stepdad ‘First time they kicked me out I was about 15, but my mum kept getting me back in’
Ian Cath	Age: 42 Gender: M Address: Street	Age of leaving school: 16 Type of school: mainstream school Performance: very poor Behaviour: good behaviour	Raised in a normal family	‘Normal childhood, went to school, passed all my exams. Joined the army, spent six years in the army. After that I was pensioned out because I had head injury in Bosnia which led to me having epilepsy. I went to France to train as a chef, I worked in the US, Hong Kong, France’	Epilepsy	Marriage breakdown ‘Evicted from flat, ended up in the street. Been homeless for a year’
Tom	Age: 36 Gender: M Address: Street	Age of leaving school: 13 Type of school: assessment centre Performance: ‘didn’t do well at school because of being abandoned child’ Behaviour: ‘expelled from school for disruptive behaviour and taking drugs and getting into crime. I was off the rails to be honest.’	Children’s home	‘I was abandoned by my mother when I was 12. I was then put into care. I was placed with my dad when I was 13, who physically abused me, then sent back to care’ ‘Life at home was chaotic, my mum and dad split up when I was eight. My dad was a very bad alcoholic and mum was very promiscuous’	Self-harm Personality disorder Severe anxiety disorder Paranoia disorder Depression	‘Slept rough on and off since I was 13’ This time we were evicted due to bedroom tax
Marie	Age: 35 Gender: F Address: Street	Age of leaving school: 13 Type of school: Mainstream Performance: poor Behaviour: challenging behaviour	Children’s care home	‘Had a bad childhood because I was sexually abused by my father when I was 13… social services knew about it because my mum’s friend rang police on him’	Migraine ‘Blackouts, hypertension and now infection in my breast due lack of hygiene’	‘Slept rough for the past three months; evicted due to bedroom tax
Leo	Age: 42 Gender: M Address: Street	Age of leaving school: 16 Type of school: Mainstream school Performance: very poor Behaviour: in trouble with school authorities	Raised by single mum	‘I left school at 16. I went to do B Tech, passed that, I went to another college, I did B Tech in National OAD, got a job at Morrison’s supermarket’ ‘I got diabetes when I was 29’ ‘I lost my dad to cancer when I was ten, my mum moved to a smaller house which was like a retirement village’	Diabetes	Evicted for missing payment due to own chaotic life’
Norma	Age: 31 Gender: F Address: Hostel for the homeless	Age of leaving school: 16 Type of school: Mainstream Performance: ‘left the school before the exams’ Behaviour: ‘disrruptive	Foster care; several children’s homes until age 6, then foster care	‘I got sexually abused by my father when I was six. So we were put into care. He abused me when I was five and raped me when I was six. Lived in foster care as well as several children’s homes from age of 6 until 16. I moved in with my sister and we have a big argument …she kicked me out and rang police on me and had [a] year[‘s] restraining order, and then I was basically in the street all the time’ ‘I have been picked up by police many times. People come up to me kick me, beat me, because I am homeless on the street’	Sleep apnoea Epilepsy Depression Self-harm Suicide attempts	‘When my two boys were taken off me and put into care, that was when my drinking spiralled out of control.’ ‘I have been in the street solidly for three years now’
Crewe	Age: 58 Gender: M Address: Flat	Age of leaving school: 15 Type of school: boarding school for pupils with emotional and behavioural difficulties Performance: very poor Behaviour: challenging behaviour	Foster care	‘My childhood was not all that good because my parents were strict. Me and my sister were separated, we were fostered out. I was fostered out with my brother to a couple in Stockport, my sisters were put into couple in Macclesfield’ ‘From 1968 to 1973 I was in boarding school. I left boarding school, started work car washing in Brookly Street and I did nine years as tyre fitter’ ‘I was put into prison at the age of 16 for arson, that was a cry for help to get away from the family. I came out after nine months. I have been in prison four times in my life, it’s not very nice, when I came out I made a promise to myself that I’m never going to go back to prison again’	Depression Self-harm Suicide attempts Angina IHD	Homeless every time he comes out of prison
Paddy	Age: 35 Gender: M Address: Hostel for homeless people Northwich	Age of leaving school: 16 Type of school: mainstream school Performance: very poor Behaviour: in trouble with school authorities	Raised by mum and stepdad until age 11	Disruptive home life, parents separated at young age. Ran away from home at the age of sixteen, and became homeless for a while until social services got involved	Depression Anxiety disorder Alcoholism Is on Librium Epilepsy	Heavy alcoholism and relationship breakdown
Matt	Age: 33 Gender: M Address: Street	Age of leaving school: 16 Type of school: local school Performance: very poor Behaviour: challeging behaviour, smoking cannibis at school	Foster care	Disruptive family life, broken family Moved in with partner at 17. Had two children, children taken into care by social services Drugs and alcohol, relationship breakdown	Alcoholism	Drugs and alcohol, relationship breakdown,
Milly	Age: 31 Gender: F address: Street	Age of leaving school: 16 Type of school: Mainstream Performance: ‘left the school before the exams’ Behaviour: ‘used to get bullied because of my abuse experience’	Put into foster care; several children’s homes until age 6, then foster care	I was in care until I was 15 and moved back with my mum and my mum die within two months of me moving in. ‘I was still in care order until I was 21’ ‘When my mum died I was out in the street drinking all the time and never stopped’	Depression Anxiety disorder Self-harm Suicide attempts	
Kieran	Age: 38 Gender: M Address: Street	Age of leaving school: 16 Type of school: Mainstream school Performance: very poor Behaviour: in trouble with school authorities	Went to children’s home at fourteen	Started using alcohol and drugs at the age of 13 Went to prison at the age of 17	Hearing voices Depression	Lost accommodation due to drug addiction

Legend: 1 illustrates participant’s education history, childhood living arrangements, brief participants’ family and social history, emotional and physical health, the onset of and trigger for homelessness

### Ethical approval

Ethical approval was obtained from the Research Ethics Committee of the University of Chester. The centre managers granted access once ethical approval had been obtained, and after their review of the study design and other research material, and of the participant information sheet which included a letter of invitation highlighting that participation was voluntary.

### Data analysis

In this study data collection and analysis occurred simultaneously. Analysis drew on Glaser’s [23] grounded theory processes of open coding, use of the constant comparative method, and the iterative process of data collection and data analysis to develop theoretical explanation of homelessness.

**Fig. 1:**
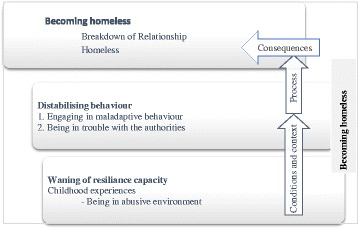
Social explanation of becoming homeless. Legend: Fig. 1 illustrates the process of becoming homeless

The process began by reading the text line-by-line identifying and open coding the significant incidents in the data that required further investigation. The findings from the initial stage of analysis are published in Mabhala [29]. The the second stage the data were organised into three themes that were considered significant in becoming homeless (see Fig. 1):

- Engaging in maladaptive behaviour
- Being in trouble with the authorities
- Being in abusive environments.

The key questions that we asked as we continued to interrogate the data were: What category does this incident indicate? What is actually happening in the data? What is the main concern being faced by the participants? Interrogation of the data revealed that participants were describing the process of becoming homeless.

The comparative analysis involved three processes described by Glaser ([23], p. 58–60): each incident in the data was compared with incidents from both the same participant and other participants, looking for similarities and differences. Significant incidents were coded or given labels that represented what they stood for, and similarly coded or labeled when they were judged to be about the same topic, theme or concept.

After a period of interrogation of the data, it was decided that the two categories - destabilising behaviour, and waning ofcapacity for resilience were sufficiently conceptual to be used as theoretical categories around which subcategories could be grouped (Fig. 1).

Once the major categories had been developed, the next step consisted of a combination of theoretical comparison and theoretical sampling. The emerging categories were theoretically compared with the existing literature. Once this was achieved, the next step was filling in and refining the poorly defined categories. The process continued until theoretical sufficiency was achieved.

## Results

Figure 1 illustrates the process of becoming homeless. The analysis revealed that becoming homeless is a process characterised by a progressive waning of resilience created by a series of adverse incidents in one’s life. Amongst the frequently cited incidents were being in an abusive environment and losing a significant person in one’s life. However, being in an abusive environment emerged from this and previously published studies as a major theme; therefore, we decided to analyse it in more detail.

The data further show that the final stage in the process of becoming homeless is a complete collapse of relationships with those with whom they live. The most prominent behaviours described by the participants as being a main cause of breakdown are:

- Engaging in maladaptive behaviour: substance misuse, alcoholism, self-harm and disruptive behaviours
- Being in trouble with the authorities: theft, burglary, arson, criminal offenses and convictions

The interrogation of data in relation to the conditions within which these behaviours occurred revealed that participants believed that their social contexts influenced their life chance, their engagement with social institution such as education and social services and in turn their ability to acquire and maintain home. Our experiences have also shown that homeless people readily express the view that behavioural lifestyle factors such as substance misuse and engaging in criminal activities are the causes of becoming homeless. However, when we spent time talking about their lives within the context of their status as homeless people, we began to uncover incidents in their lives that appeared to have weakened their capacity to constructively engage in relationships, engage with social institutions to make use of social goods [29–31] and maturely deal with societal demands.

### Being in abusive environments

Several participants explicitly stated that their childhood experiences and damage that occurred to them as children had major influences on their ability to negotiate their way through the education system, gain and sustain employment, make appropriate choices of social networks, and form and maintain healthy relationships as adults.

It appears that childhood experiences remain resonant in the minds of homeless participants, who perceive that these have had bearing on their homelessness. Their influence is best articulated in the extracts below. When participants were asked to tell their stories of what led to them becoming homeless, some of their opening lines were:

*What basically happened, is that I had a childhood of so much persistent, consistent abuse from my mother and what was my stepfather. Literally consistent, we went around with my mother one Sunday where a friend had asked us to stay for dinner and mother took the invitation up because it saved her from getting off her ass basically and do anything. I came away from that dinner genuinely believing that the children in that house weren’t loved and cared for, because they were not being hit, there was no shouting, no door slamming.* [Marco]

It appears that Marco internalised the incidents of abuse, characterised by shouting, door slamming and beating as normal behaviour. He goes on to intimate how the internalised abusive behaviour affected his interaction with his employers.

*‘…but consistently being put down, consistently being told I was thick, I started taking jobs and having employers effing and blinding at me. One employer actually used a “c” word ending in “t” at me quite frequently and I thought it was acceptable, which obviously now I know it’s not. So I am taking on one job after another that, how can I put it? That no one else would do basically. I was so desperate to work and earn my own money.* [Marco]

Similarly, David makes a connection between his childhood experience and his homelessness. When he was asked to tell his life story leading to becoming homeless, his opening line was:

*I think it [homelessness] started off when I was a child. I was neglected by my mum. I was physically and mentally abused by my mum. I got put into foster care, when I left foster care I was put in the hostel, from there I turn into alcoholic. Then I was homeless all the time because I got kicked out of the hostels, because you are not allowed to drink in the hostel.* [David]

David and Marco’s experiences are similar to those of many participants. The youngest participant in this study, Clarke, had fresh memories of his abusive environment under his stepdad:

*I wouldn't want to go back home if I had a choice to, because before I got kicked out me stepdad was like hitting me. I wouldn't want to go back to put up with that again.*

*[I didn't tell anyone] because I was scared of telling someone and that someone telling me stepdad that I've told other people. ‘[Be] cause he might have just started doing again because I told people. It might have gotten him into trouble.* [Clarke]

In some cases, participants expressed the beliefs that their abusive experience not only deprived them life opportunities but also opportunities to have families of their own. As Tom and Marie explain:

*We were getting done for child neglect because one of our child has a disorder that means she bruise very easily. They all our four kids into care, social workers said because we had a bad childhood ourselves because I was abused by my father as well, they felt that we will fail our children because we were failed by our parents. We weren’t given any chance* [Tom and Marie]

Norma, described the removal of her child to care and her maladaptive behaviour of excessive alcohol use in the same context as her experience of sexual abuse by her father.

*I had two little boys with me and got took off from me and put into care. I got sexually abused by my father when I was six. So we were put into care. He abused me when I was five and raped me when I was six. Then we went into care all of us I have four brothers and four sisters. My dad did eighteen months for sexually abusing me and my sister. I thought it was normal as well I thought that is what dads do* [Norma]

The analysis of participants in this study appears to suggest that social condition one is raised influence the choice of social connections and life partner. Some participants who have had experience of abuse as children had partner who had similar experience as children Tom and Marie, Lee, David and his partners all had partners who experienced child abuse as children.

Tom and Marie is a couple we interviewed together. They met in hostel for homeless people they have got four children. All four children have been removed from them and placed into care. They sleep rough along the canal. They explained:

*We have been together for seven years we had a house and children social services removed children from us, we fell within bedroom tax. …we received an eviction order …on the 26th and the eviction date was the 27th while we were in family court fighting for our children. …because of my mental health …they were refusing to help us.*

*Our children have been adopted now. The adoption was done without our permission we didn’t agree to it because we wanted our children home because we felt we were unfairly treated and I [Marie] was left out in all this and they pin it all on you [Tom] didn’t they yeah, my [Tom] history that I was in care didn’t help.*

Tom went on to talk about the condition under which he was raised:

*I was abandoned by my mother when I was 12 I was then put into care; I was placed with my dad when I was 13 who physically abused me then sent back to care.* [Tom].

David’s story provides another example of how social condition one is raised influence the choice of social connections and life partner. David has two children from two different women, both women grew up in care. Lisa one of David’s child mother is a second generation of children in care, her mother was raised in care too.

*I drink to deal with problems. As I say I’ve got two kids with my girlfriend Kyleigh, but I got another lad with Lisa, he was taken off me by social services and put on for adoption ten years ago and that really what started it; to deal with that. Basically, because I was young, and I had been in care and the way I had been treated by my mum. Basically laid on me in the same score as my mum and because his mum [Lisa] was in care as well. So they treated us like that, which was just wrong.* [David]

### Engaging in maladaptive behaviour

In this study, most participants identified alcohol or drugs and crime as the cause of relationships breakdown. However, the language they used indicates that these were secondary reasons rather than primary reasons for their homelessness. The typical question that MA and MG asked the interview participants was “tell us how did you become homeless”? Typically, participants cited different maladaptive behaviours to explain how they became homeless.

Alvin’s story is typical of:

*Basically I started off as a bricklayer, … when the recession hit, there was an abundance of bricklayers so the prices went down in the bricklaying so basically with me having two young children and the only breadwinner in the family... so I had to kinda look for factory work and so I managed to get a job… somewhere else…. It was shift work like four 12 hour days, four 12 hour nights and six [days] off and stuff like that, you know, real hard shifts.*

*My shift was starting Friday night and I’ll do Friday night, Saturday night to Monday night and then I was off Tuesday, Wednesday and Thursday, but I’d treat that like me weekend you know because I’ve worked all weekend. Then… so I’d have a drink then and stuff like that, you know. 7 o’ clock on a Monday morning not really the time to be drinking, but I used to treat it like me weekend.*

*So we argued, me and my ex-missus [wife], a little bit and in the end we split up so moved back to me mum's, but kept on with me job, I was at me mum’s for possibly about five years and but gradually the drinking got worse and worse, really bad. I was diagnosed with depression and anxiety.*

*… I used to drink to get rid of the anxiety and also to numb the pain of the breakup of me marriage really, you know it wasn’t good, you know. One thing led to another and I just couldn’t stop me alcohol. I mean I’ve done drugs you know, I was into the rave scene and I’ve never done hard drugs like heroin or... I smoke cannabis and I use cocaine, and I used to go for a pint with me mates and that.*

*It all came to a head about November/December time, you know it was like I either stop drinking or I had to move out of me mum's. I lost me job in the January through being over the limit in work from the night before uum so one thing led to another and I just had to leave. [Alvin]*

Similarly, Gary identified alcohol as the main cause of his relationship breakdown. However, when one listens to the full story alcohol appears to be a manifestation of other issues, including financial insecurities and insecure attachment etc.

*It [the process of becoming homeless] mainly started with the breakdown of the relationship with me partner. I was with her for 15 years and we always had somewhere to live but we didn't have kids till about 13 years into the relationship. The last two years when the kids come along, I had an injury to me ankle which stopped me from working. I was at home all day everyday. …I was drinking because I was bored. I started drinking a lot ‘cause I couldn't move bout the house. It was a really bad injury I had to me ankle. Um, and one day me and me partner were having this argument and I turned round and saw my little boy just stood there stiff as a board just staring, looking at us. And from that day on I just said to me partner that I'll move out, ‘cause I didn't want me little boy to be seeing this all the time.* [Gary]

In both cases Gary and Alvin indicate that changes in their employment status created conditions that promoted alcohol dependency, though both explained that they drank alcohol before the changes in their employment status occurred and the breakdown of relationships. Both intimated that that their job commitment limited the amount of time available to drink alcohol. As Gary explained, it is the frequency and amount of alcohol drinking that changed as a result of change in their employment status:

*I used to have a bit of a drink, but it wasn’t a problem because I used to get up in the morning and go out to work and enjoy a couple of beers every evening after a day’s work. Um, but then when I wasn't working I was drinking, and it just snowballed out, you know snowball effect, having four cans every evening and then it went from there. I was drinking more ‘cause I was depressed. I was very active before and then I became like non-active, not being able to do anything and in a lot of pain as well.* [Gary]

Furthermore, although the participants claim that drinking alcohol was not a problem until their employment circumstances changed, one gets a sense that alcohol was partly responsible for creating conditions that resulted in the loss of their jobs. In Gary’s case, for example, alcohol increased his vulnerability to the assault and injuries that cost him his job:

*I got assaulted, kicked down a flight of stairs. I landed on me back on the bottom of the stairs, but me heel hit the stairs as it was still going up if you know what I mean. Smashed me heel, fractured me heel… So, by the time I got to the hospital and they x-rayed it they wasn't even able to operate ‘cause it was in that many pieces, they weren't even able to pin it if you know what I mean.* [Gary]

Alvin, of the other hand, explained that:

*I lost my job in the January through being over the limit in work from the night before, uum so one thing led to another and I just had to leave.* [Alvin]

In all cases participants appear to construct marriage breakdown as an exacerbating factor for their alcohol dependence. Danny, for example, constructed marriage breakdown as a condition that created his alcohol dependence and alcohol dependence as a cause of breakdown of his relationship with his parents. He explains:

*I left school when I was 16. Straight away I got married, had children. I have three children and marriage was fine. Umm, I was married for 17 years. As the marriage broke up I turned to alcohol and it really, really got out of control. I moved in with my parents... It was unfair for them to put up with me; you know um in which I became... I ended up on the streets, this was about when I was 30, 31, something like that and ever since it's just been a real struggle to get some permanent accommodation.* [Danny]

Danny goes on to explain:

*Yes [I drank alcohol before marriage broke down but] not very heavily, just like a sociable drink after work. I'd call into like the local pub and have a few pints and it was controlled. My drinking habit was controlled then.*

*I did go back to my parents after my marriage break up, yes. I was drinking quite heavily then. I suppose it was a form of release, you know, in terms of the alcohol which I wish I'd never had now.*

*When I did start drinking heavy at me parents’ house, I was getting in trouble with the police being drunk and disorderly. That was unfair on them.* [Danny]

The data in this study indicate that homelessness occurs when the relationships collapse, irrespective of the nature of the relationship. There were several cases where lifestyle behaviour led to a relationship collapse between child and parents or legal guardians.

In the next excerpt, Emily outlines the incidents: smoking weed, doing crack and heroin, and drinking alcohol. She also uses the words ‘because’, ‘when’ and ‘obviously’, which provide clues about the precipitating condition for her behaviours “spending long time with people who take drugs”.

I've got ADHD like, so obviously my mum kicked me out when I was 17 and then like I went to **Beswick** and stuff like that.

*My mum in the end just let me do what I wanted to do, ‘cause she couldn't cope anymore. …I mean I tried to run away from home before that, but she'd always like come after me in like her nightie and pyjamas and all that. But in the end she just washed her hands of me*. [Emily]

Emily presented a complex factors that made it difficult for her mother to live with her. These included her mother struggle with raising four kids as a single parent, Emily’s mental health (ADHD], alcohol and drug use. She goes on to explain that:

*Ummm, well the reason I got kicked out of my hostel was ‘cause of me drinking, so I'd get notice to quit every month, then I’d have a meeting with the main boss and then they'd overturn it and this went on every month for about six months. Also, it was me behaviour as well, but obviously drink makes you do stuff you don't normally do and all that shit.*

*I lived here for six months, got kicked out because I jumped out the window and broke me foot. I was on the streets for six months and then they gave me a second chance and I've been here a year now. So that's it basically.* [Emily]

There were several stories of being evicted from accommodation due to excessive use of alcohol. One of those is David:

*I got put into foster care. When I left foster care I was put in the hostel, from there I turn into alcoholic. Then I was homeless all the time because I got kicked out of the hostels, because you are not allowed to drink in the hostel. It’s been going on now for about… I was thirty-one on Wednesday, so it’s been going on for about thirteen years, homeless on and off. Otherwise if not having shoplifted for food and then go to jail, and when I don’t drink I have lot of seizures and I end up in the hospital. Every time I end up on the street.*

*I trained as a chef, I have not qualified yet, because of alcohol addiction, it didn’t go very well. I did couple of jobs in restaurants and diners, I got caught taking a drink.* [David]

Contrary to the other incidents where alcohol was a factor that led to homelessness, Barry’s description of his story appears to suggest that the reason he had to leave his parents’ home was his parents’ perception that his sexuality brought shame to the family:

*When I came out they I’m gay, my mum and dad said you can’t live here anymore. I lived in a wonderful place called Nordic... but fortunately, mum and dad ran a pub called […] [and] one of the next door neighbours lived in a mansion. His name was [….] [and] when I came out, he came out as in he said “I'm a gay guy”, but he took me into Liverpool and housed me because I had nowhere to live. My mum and dad said you can't live here anymore.*

*And unfortunately, we get to the present day. I got attacked. I got mugged... only walked away with a £5 note, it’s all they could get off me. They nearly kicked me to death so I was in hospital for three weeks. By the time I came out, I got evicted from my flat. I was made homeless.* [Barry]

We used the phrase “engaging in maladaptive behaviour” to conceptualise the behaviours that led to the loss of accommodation because our analysis appear to suggest that these behaviours were strategies to cope with the conditions they found themselves in. For example, all participants in this category explained that they drank alcohol to cope with multiple health (mental health) and social challenges.

### Being in trouble with the authorities

In the UK adulthood homelessness is more visible than childhood homelessness. However, most participants in this research reveal that the process of becoming homeless begins at their childhood, but becomes visible after the legal age of consent (16). Participants described long history of trouble with people in authority including parents, legal guardians and teachers. However, at the age of 16 they gain legal powers to leave children homes, foster homes, parental homes and schools, and move outside some of the childhood legal protections. Their act of defiance becomes subject to interdiction by the criminal justice system. This is reflected in number of convictions for criminal offenses some of the participants in this study had.

Participants Ruddle, David, Lee, Emily, Pat, Marco, Henry and many other participants in this study (see Table 1) clearly traced the beginning of their troubles with authority back at school. They all expressed the belief that had their schooling experience been more supportive, their lives would have been different. Lee explains that being in trouble with the authorities began while he was at school:

*‘The school I came from a rough school, it was a main school, it consisted of A, B, C, D and The school I came from [was] a rough school, it was a main school, it consisted of A, B, C, D and E. I was in the lowest set, I was in E because of my English and maths. I was not interested, I was more interested in going outside with big lads smoking weed, bunking school. I used to bunk school inside school. I used to bunk where all cameras can catch me.*

*They caught me and reported me back to my parents. My mum had a phone call from school asking where your son is. My mum grounded me. While my mum grounded me I had a drain pipe outside my house, I climbed down the drain pipe outside my bedroom window. I used to climb back inside.* [Lee]

Lee’s stories constructed his poor education experiences as a prime mover towards the process of becoming homeless. It could be noted in Table 1 that most participants who described poor education experiences came from institutions such as foster care, children home and special school for maladjusted children. These participants made a clear connection between their experiences of poor education characterised by defiance of authorities and poor life outcomes as manifested through homelessness.

Patrick made a distinct link between his school experience and his homelessness, for example, when asked to tell his story leading up to becoming homeless, Patrick’s response was:

*I did not go to school because I kept on bunking. When I was fifteen I left school because I was caught robbing. The police took me home and my mum told me you’re not going back to school again, you are now off for good. Because if you go back to school you keep on thieving, she said I keep away from them lads. I said fair enough. When I was seventeen I got run over by a car.* [Patrick]

Henry traces the beginning of his troubles with authorities back at school:

*[My schooling experience]… was good, I got good, well average grades, until I got myself into [a] few fights mainly for self-defence. In primary schools, I had a pretty... I had a good report card. In the start of high school, it was good and then when the fights started that gave me sort of like a... bad reputation. I remember my principal one time made me cry. Actually made me cry, but eh...*

*I don't know how, but I remember sitting there in the office and I was crying. My sister also stuck up for me when she found out what had happened, she was on my side; but I can’t remember exactly what happened at that time.* [Henry]

Emily’s story provides some clues about the series of incidents - including, delay in diagnosing her health condition, being labelled as a naughty child at school, being regularly suspended from school and consequently poor educational attainment.

*Obviously, I wasn't diagnosed with ADHD till I was like 13, so like in school they used to say that's just a naughty child. … So it was like always getting suspended, excluded and all that sort of stuff. And in the end [I] went to college and the same happened there.* [Emily]

The excerpt above provides intimations of what she considers to be the underlying cause of her behaviour towards the authorities. Emily suggests that had the authorities taken appropriate intervention to address her condition, her life outcomes would have been different.

Although the next participant did not construct school as being a prime mover of their trouble with authorities, their serious encounters with the criminal justice system occurred shortly after leaving school:

*Well I did a bit of time at a very early age, I was only 16… I did some remand there, but then when I went to court ‘cause I'd done enough remand, I got let out and went to YMCA in Runcorn. Well, that was when I was a kid. When I was a bit older, ‘cause it was the years 2000 that I was in jail, I was just trying to get by really. I wasn’t with Karen at the time. I was living in Crewe and at the time I was taking a lot of amphetamines and was selling amphetamines as well, and I got caught and got a custodial sentence for it. But I've never been back to jail since. I came out in the year 2000 so it's like 16 years I've kept meself away from jail and I don't have any intentions of going back.* [Gary]

The move from school and children social care system to criminal justice was a common pathways for many participants in this study. Some including Lee, Crewe, David, Patrick spent multiple prison sentences (see Table 1). Although Crewe did not make connection between his schooling experiences and his trouble with law, it could be noted that his serious encounter with criminal justice system started shortly after leaving foster care and schooling systems. As he explains:

*I was put into prison at age of 17 for arson that was a cry for help to get away from the family, I came out after nine months. I have been in prison four times in my life, its not very nice, when I came out I made a promise to myself that I’m never going to go back to prison again. *[Crewe]

Lee recalls his education experience. He explained:

*I left school when I was fifteen… then I went off the rails. I got kidnapped for three and half months. When I came back I was just more interested in crime. When I left school I was supposed to go to college, but I went with travellers. I was just more interested in getting arrested every weekend, until my mum say right I have enough of you. I was only seventeen. I went through the hostels when I was seventeen.* [Lee]

None describe the educational experience with a similar profundity to Marco:

*On few occasions I came out on the corridors I would be getting battered on to my hands and knees and teachers walk pass me. There was quite often blood on the floor from my nose, would be punched on my face and be thrown on the floor. …. It was hard school, pernicious. I would go as far as saying I never felt welcome in that school, I felt like a fish out of the water, being persistently bullied did my head in.*

*Eventually I started striking back, when I started striking back suddenly I was a bad one. My mother decided to put me in … school for maladjusted boys, everyone who been there including myself have spent time in prison.* [Marco]

The trouble with authorities that was observes in participants stories in this category appear to be part of the wider adverse social challenges that the participants in this study were facing. Crewe’s description of arson as a cry for help appears to be an appropriate summation of all participants in this category.

The participants’ description of the social conditions in which were raised and their references to maladaptive behaviours which led to them becoming homeless, led us to conclude that they believe that their social condition affected their life chances: that these conditions were responsible for their low quality of social connections, poor educational attainment, insecure employment and other reduced life opportunities available to them.

## Discussion

The key feature that distinguish this study from comparable previous studies is that it openly acknowledges that data collection and analysis were influenced by the principles of social justice [28, 30, 31]. The resulting theoretical explanation therefore constitutes our interpretation of the meanings that participants ascribe to their own situations and actions in their contexts. In this study, defining homelessness within the wider socioeconomic context seemed to fit the data, and offered one interpretation of the process of becoming homeless.

While the participants’ experiences leading to becoming homeless may sound trite. What is pertinent in this study is understanding the conditions within which their behaviours occurred. The data were examined through the lens of social justice and socio-economic inequalities: we analysed the social context within which these behaviours occurred. We listened to accounts of their schooling experiences, how they were raised and their social network. The intention was not to propose a cause-and-effect association, but to suggest that interventions to mitigate homelessness should consider the social conditions within which it occurred.

Participants in this study identified substance misuse and alcohol dependency as a main cause of their homelessness. These findings are consistent with several epidemiological studies that reported a prevalence of substance misuse amongst the homeless people [32–36]. However, most these studies are epidemiological; and by nature epidemiological studies are the ‘gold standard’ in determining causes and effects, but do not always examine the context within which the cause and effect occur. One qualitative study that explored homelessness was a Canadian study by Watson, Crawley and Cane [37]. Participants in the Watson, et al. described ‘lack of quality social interactions and pain of addition. However, Watson et al. focus on the experiences of being homeless, rather than the life experiences leading to becoming homeless. To our knowledge the current study is one of very few that specifically examine the conditions within which homelessness occurs, looking beyond the behavioural factors. Based on the synthesis of data from previous studies, it makes sense that many interventions to mitigate homelessness focus more on tackling behavioural causes of homelessness rather than fundamental determinants of it [38]. From the public health intervention’ point of view, however, understanding the conditions within which homelessness occurs is essential, as it will encourage policymakers and providers of the services for homelessness people to devote equal attention to tackling the fundamental determinants of homelessness as is granted in dealing behavioural causes.

Participants in this study reported that they have been defiant toward people in positions of authority. For most of them this trouble began when they were at school, and came to the attention of the criminal justice system as soon as they left school at the age of 16. These findings are similar to these in the survey conducted by Williams, Poyser, and Hopkins [39] which was commissioned by the UK Ministry of Justice. This survey found that 15 % of prisoners in the sample reported being homeless before custody [39]; while three and a half percent of the general population reported having ever been homeless [39]. As the current study reveals there are three possible explanations for the increased population of homeless young people in the criminal justice system: first, at the age of 16 they gain legal powers to leave their foster homes, parents homes, and schools and move beyond some of the childhood legal protections; second, prior to the age of 16 their defiant behaviours were controlled and contained by schools and parents/legal guardians; and third, after the age of 16 their acts of defiant behaviour become subject to interdiction by the criminal justice system.

The conditions in which they were born and raised were described by some participants in this study as ‘chaotic’, abusive’, ‘neglect’, ‘pernicious’ ‘familial instability’, ‘foster care’, ‘care home’, etc. Taking these conditions, and the fact that all but one participants in this left school at or before the age of 16 signifies the importance of living conditions in educational achievement. It has been reported in previous studies that children growing up in such conditions struggle to adjust in school and present with behavioural problems, and thus, poor academic performance [40]. It has also been reported that despite these families often being known to social services, criminal justice systems and education providers, the interventions in place do little to prevent homelessness [40].

Analysis of the conditions within which participants’ homelessness occurred reveals the adverse social conditions within which they were born and raised. The conditions they described included being in an abusive environment, poor education, poor employment or unemployment, poor social connections and low social cohesion. These conditions are consistent with high index of poverty [37, 41, 42]. And several other studies found similar associations between poverty and homelessness [42]. For example, the study by Watson, Crowley et al. [37] found that there were extreme levels of poverty and social exclusion amongst homeless people. Contrary to previous studies that appear to construct homelessness as a major form of social exclusion, the analysis of participants’ stories in this current study revealed that the conditions they were raised under limited their capacity to engage in meaningful social interactions, thus creating social exclusion.

## Conclusion

Homeless people describe the immediate behavioural causes of homelessness; however, this analysis revealed the social and economic conditions within which homelessness occurred. The participants’ descriptions of the social conditions in which were raised and their references to maladaptive behaviours which led to them becoming homeless, led us to conclude that they believe that their social condition affected their life chances: that these conditions were responsible for their low quality of social connections, poor educational attainment, insecure employment and other reduced life opportunities available to them.

## Limitations

The conclusions drawn relate only to the social and economic context of the participants in this study, and therefore may not be generalised to the wider population; nor can they be immediately applied in a different context. It has to be acknowledged that the method of recruitment of the 26 participants generates a bias in favour of those willing to talk. The methodology used in this study (constructivist grounded theory) advocates mutual construction of knowledge, which means that the researchers’ understanding and interpretations may have had some influence on the research process as the researchers are an integral part of the data collection and analysis
